# Investigating populations via penguins and their poo!

**DOI:** 10.1111/test.12195

**Published:** 2019-02-27

**Authors:** Laura J. Bonnett, Simon R. White

**Affiliations:** ^1^ Department of Biostatistics University of Liverpool Liverpool England; ^2^ MRC Biostatistics Unit University of Cambridge Cambridge England

**Keywords:** Teaching, populations, practical activity, samples, teaching statistics

## Abstract

We describe an activity that introduces students to population modelling, enables them to use estimates obtained from a sample to infer back to the population, and understands how the findings are translatable via penguins and their poo!

## 1 INTRODUCTION

It is likely that most secondary school students (aged 11‐18 y) are familiar with the colloquial definition of a population. For example, if you ask them what the population of their country is, it is probable that they understand the question to mean how many people live there. In the authors' experience, a question regarding what is a sample is frequently answered relating to free food in supermarkets! Although neither answer is incorrect, the statistical definitions of populations and samples are vital for many aspects of statistical methodology and understanding and thus we should ensure careful definition of these terms. One way to ensure students are familiar with the statistical definition of the terms population and sample is via engaging activities at science festival exhibitions.

Science festival exhibitions and fairs offer an invaluable opportunity to engage school students with statistics. Colorful, appealing activities attract people to a stall and enable demonstrations of a statistical concept in less than 5 minutes. Feedback received by the authors to date on their previous stalls suggests that the most popular activities are those involving animals and perceived unsavory topics such as poo. Despite the taboo and disgust often associated with fecal matter 1, children and adults of all ages are still fascinated by it 2 making it a particularly useful hook for an activity! This paper uses the informal term “poo” rather than other euphemisms, as this is more suitable terminology for the audience at science fairs or festivals.

We therefore propose a novel activity to demonstrate populations and samples via penguin toys and a photograph of penguin poo on the snow. This activity is inspired by research conducted on the Emperor penguin population, as reported in a Significance magazine article in 2012 3. In that article, Peter Fretwell reported on advances in understanding the scale and nature of penguin populations through satellite imagery and commented “the race is on to calculate the population.” His solution was to use new, higher‐resolution imagery to count small squares (pixels) that were black, representing the area covered by penguin bodies, and use a sample of on‐the‐ground surveys to estimate the number of penguins in those black squares 4.

The learning aim of our novel activity is to understand the concepts of populations and samples and why that can be useful in real‐life applications. Our activity can be tailored to the available time, context, and level of the target audience. In the following sections, we present a suggested template for delivering the activity at a science festival exhibition or similar for single groups of students over a very short duration of time and as a classroom version with increased capacity and duration.

## 2 MATERIALS

**Figure 1:**
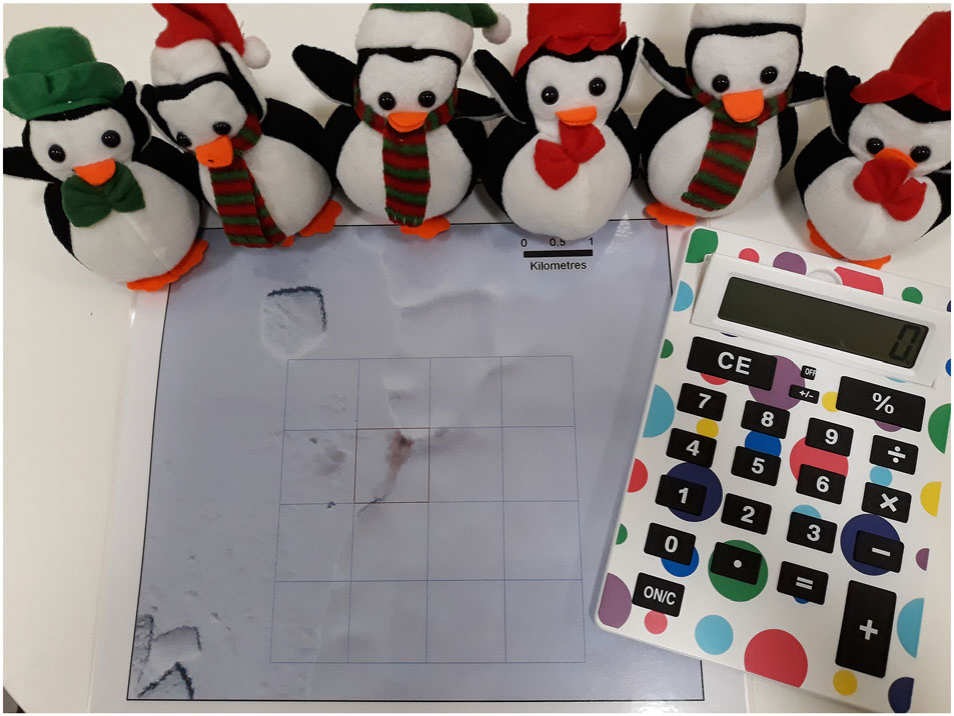
Required resources—penguins, calculator, and photograph of penguin poo on snow [Colour figure can be viewed at wileyonlinelibrary.com]

Our activity requires approximately 12 toy penguins (or images of penguins) that can be lifted and replaced with ease, a calculator, the bigger the better, and an aerial photograph of penguin poo on snow with an associated scale, as shown in Figure 1. An appropriate scaled photograph of the penguin poo is available to download from www.rss.org.uk/hands‐on.

The penguins and calculator are fairly easy to source ensuring that the activity is ideal for science festivals. The classroom‐based version of this activity can be run using images of penguins rather than toys to ensure minimal costs—indeed, it is possible to encourage students to make penguins in advance of the activity as a cross‐curricular activity.

## 3 THE ACTIVITY

**Figure 2:**
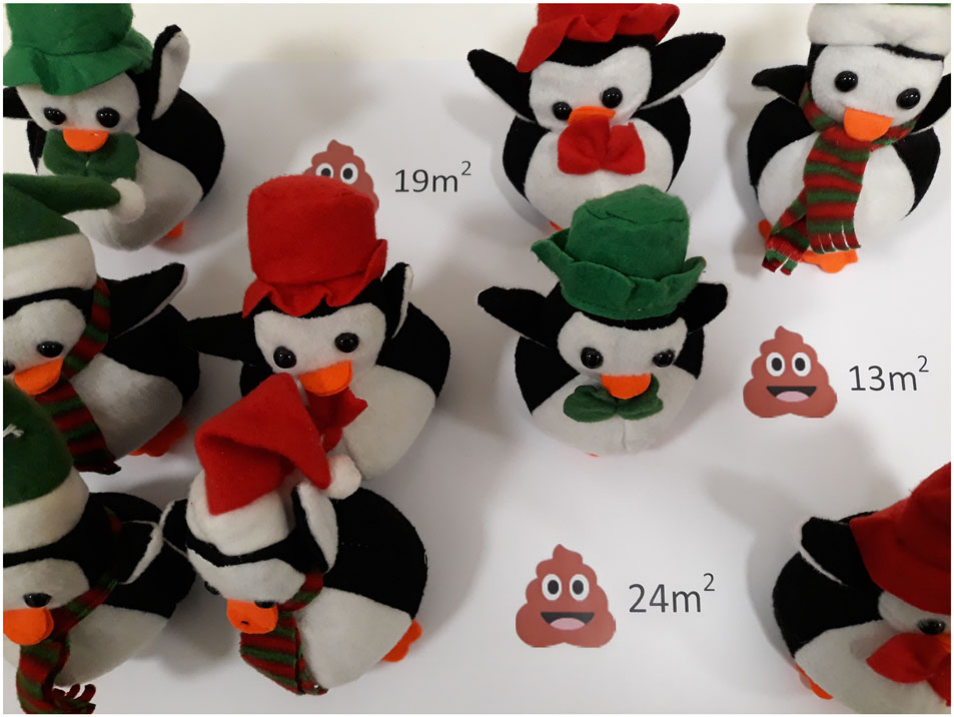
Toy penguins sat on a sheet of paper that describes the annual amount of poo produced per penguin [Colour figure can be viewed at wileyonlinelibrary.com]

Prior to the event, decide on the annual amount of poo produced per toy penguin via a sticker either on the base of each penguin or on a sheet of paper that the penguins sit on (as in Figure 2). As a guide, it is estimated that there are approximately 10 000 penguins per colony 3. The downloadable scale photograph shows approximately 150 000 m^2^ of poo. Consequently, the mean poo produced by the toy penguins should be in the region of 15 m^2^. The authors generated uniform random numbers between 5 and 25 m^2^ (rounded to the nearest integer) for the sheet shown in Figure 2. For primary school students, priority should be given to numerical simplicity over biological accuracy. Therefore, values of 1 to 4 m^2^ are recommended as these enable calculation of the total sample poo via mental arithmetic, with the use of fingers as necessary.

The first element of this activity requires a discussion of the aerial photograph—this is an activity about penguins, and penguins live on the snow in Antarctica. What can the student see on the snow? Distinct penguin shapes? At the provided resolution, it is impossible to see and thus count the number of penguins in the photograph.

Next, we explain that the brown patch visible on the snow is actually penguin poo and that working out how much poo is in the photo will enable us to work out how many penguins live in the area of snow captured by the photograph. We then explain the related statistical term—a population. The population of interest in this activity is all the penguins producing the brown patch visible in the aerial photograph.

Next, we help students estimate the area of the poo produced using the provided scale (or specify it for younger participants). Surface area in units squared (km^2^ according to the suggested photograph) can be obtained by multiplying the horizontal dimension of the poo by the vertical dimension of the poo. We then explain that if we know how much poo one penguin produces, we can divide the total surface area by the amount of poo for one penguin to get an approximation of the number of penguins producing the poo in the photograph.

To establish how much poo a penguin produces, we encourage students to consider locations with more readily available penguins than those living in Antarctica. Many suggest sea life centers and zoos with little prompting. At this point, the participants should be introduced to the toy penguins, which are to become the local penguins for the purposes of this activity, and a secondary population of interest. It is important to explain to students that underneath each toy is an estimate of the amount of poo produced per year per penguin.

A discussion should then take place regarding the reliability of information from a single penguin. If the chosen penguin produces a very small amount of poo in comparison with the other penguins, for example, this would lead to an overestimate of the number of penguins in the colony shown in the photograph. Therefore, it is more appropriate to calculate the mean amount of poo produced by several penguins.

A student should then be encouraged to select three penguins from the collection of toy penguins and lift them up to reveal the amount of poo produced by the sampled penguins. A random sample of penguins could be obtained by use of a random number generator. However, for the purposes of this activity, students are just encouraged to pick any three penguins. Next, explain to students that the three selected penguins are a sample of all the toy penguins and that they can be used to estimate the mean amount of poo from the sampled toy penguins. Students should then be assisted to calculate the mean amount of poo using the formula:

$$\text{Sample mean}\;\mathrm{poo}=\frac{\text{Penguin}\;1\;\mathrm{poo}+\;\text{Penguin}\;2\;\mathrm{poo}+\text{Penguin}\;3\;\mathrm{poo}}{3}.$$

From this sample mean poo, which is described as a statistic in statistical terminology, we help the participant(s) to estimate the number of penguins in the aerial photograph and hence in that colony of penguins within Antarctica by using the second formula:

$$\text{Estimated number of penguins in the population}=\frac{\text{Surface area of}\;\mathrm{poo}\;\text{in photo}}{\text{Mean}\;\mathrm{poo}\;\text{of sampled}\;\mathrm{toy}\;\text{penguins}}.$$

We conclude this activity by explaining that statistics calculated from any sample can be used to infer information about the associated population. Therefore, in this activity, the sample mean poo was used to estimate the number of penguins in the population producing the poo visible in the photograph.

## 4 CLASSROOM EXTENSION

In the science festival exhibition version, we encouraged students to pick a single sample of three toy penguins and estimate their mean amount of poo and then to use that statistic to estimate the number of penguins living in the colony seen in the aerial photograph. This naturally leads to several questions that can be addressed in a classroom extension of the activity. For example, what happens if a different sample of toy penguins is selected? What about if a sample of five or 10 toy penguins are used? What happens if the sample of toy penguins is not randomly selected?

To extend the science festival exhibition version, and to answer these questions, we can encourage a class to undertake a simulation study—a way of considering what happens over multiple interactions with the activity. Therefore, in the simplest extension, a class should be split into groups of two or three students, each with a set of about 12 penguins. Each group should then be encouraged to estimate the number of penguins producing the brown patch in the snow using the methodology described in science festival exhibition version of the activity. This will lead to multiple different estimates of the sample mean amounts of poo across the class and thus multiple different estimates of the number of penguins in the photograph across the class. Students should be encouraged to note that different random samples lead to different estimates of the mean poo produced and thus different numbers of penguins producing the poo shown in the aerial photograph.

In the second extension, students will consider the effect of different sample sizes. First, toy penguins from each group should be merged into one large population of toy penguins. Next, the students should establish how many penguins are producing the poo in the photo by estimating the mean amount of poo produced by the entire population of toy penguins. Of course, in reality, this estimate is unknown due to the infeasibility of sampling everyone in the population. However, it is important for this extension to be able to compare the results of the simulation study with the results for the entire population of toy penguins.

**Figure 3:**
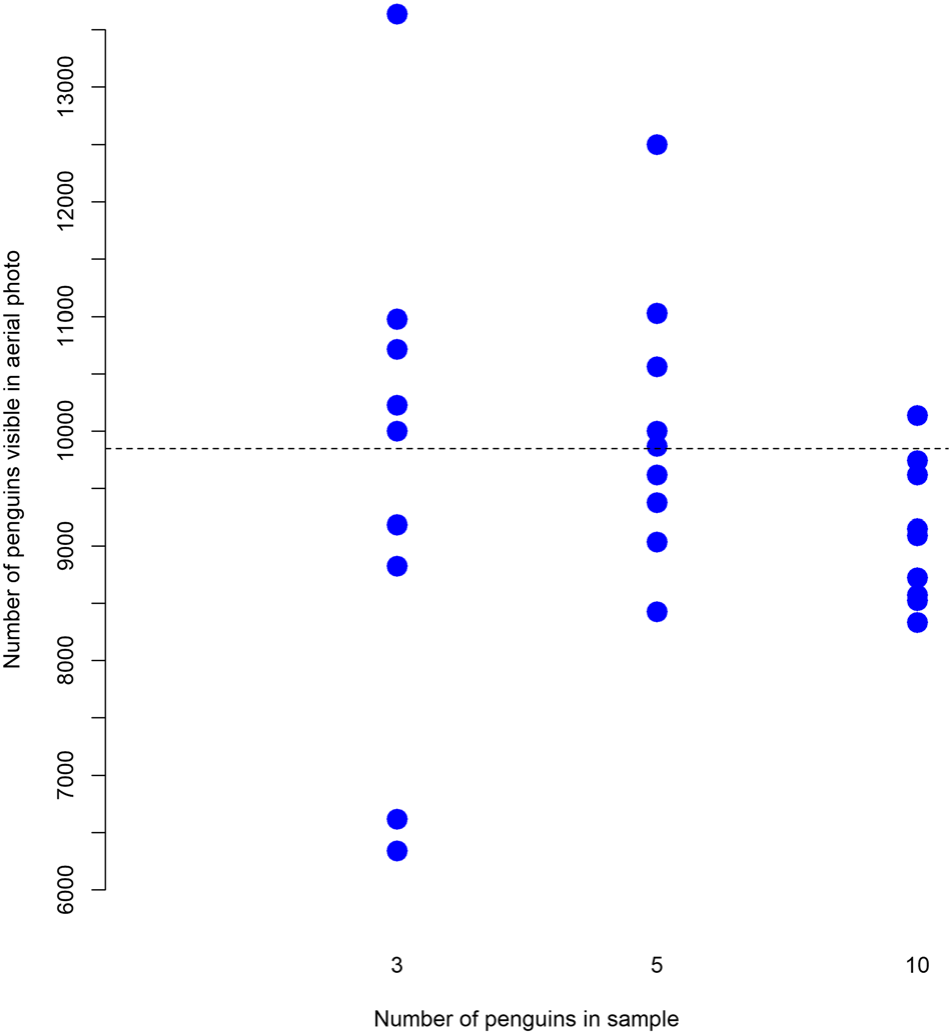
Plot of results from simulation studies—dotted line represents the estimated number of penguins seen in the photograph based on the mean poo of all the toy penguins [Colour figure can be viewed at wileyonlinelibrary.com]

Small groups of students should each then take a sample of toy penguins from the entire population of toy penguins but this time of five penguins and then 10 penguins. Students should then estimate the mean amount of poo produced from their sampled penguins and thus estimate the number producing the poo in the photograph. The results from each group should then be plotted as shown in Figure 3, to include the results for samples of three penguins from the first extension. This should highlight that the samples do in fact vary around the “true number” and that the larger the sample, the smaller this variability will be.

The final extension can be used to investigate the effect of nonrandom sampling. For this extension, we suggest that the students modify the penguins in some way so that there are at least two groups of penguins—the penguins we use are already modified with red and green colored hats and scarves as shown in Figures 1 and 2. Once modified, encourage students to arrange the toy penguins such that those with scarves (or equivalent) produce smaller amounts of poo per year than those without for example. (We avoid separating by red and green to ensure the activity is inclusive for people who are color‐blind.)

Begin the extension by asking each group of students to pick five penguins from all toy penguins, regardless of scarf status. Record the sample mean poo, and plot the resulting estimates of the number of penguins producing the photographed poo for the class. Then ask each group to pick five of only those penguins in scarves and add the results in a colored pen or similar. Finally, ask each group to pick five of only those penguins without scarves and plot the results in a different color. This might be equivalent to sampling rockhopper penguins when those in the photograph are known to be Emperor penguins for example. Encourage students to consider the implications of this—namely, that we must be aware of the population of interest and the sample we are taking.

## 5 CONCLUSION

Populations and samples are fairly familiar concepts within general conversation. However, their use within statistics is potentially unfamiliar despite being essential concepts. A basic understanding of sampling has many real‐life applications. For example, a random selection of items will be checked prior to leaving a factory to ensure that they are fit for purpose 5, 6. Alternatively, counting red squirrels in a random grid square enables us to assess numbers living in a particular forest for example 7. We therefore propose a novel way of considering population modelling that can be utilized as a quick‐hitting activity at a science fair, or as a more in‐depth classroom activity. The method described in this activity is used in reality, and thus, the activity could run in conjunction with a science class.

The science festival exhibition version and all associated extensions of the activity introduce students to the concept of populations and samples via penguin poo! Students are encouraged to calculate a statistic from a random sample that they then use to infer the size of the population with various possible variations. In particular, they are required to think about the size of the sample, the number of samples to take, and the mix of sampled items. By demonstrating to students that a small sample of readily available penguins can be used to estimate how many live in a colony in Antarctica, we hope to convince students of the real‐life potential of statistics.
